# Diphtheria, Scarlet Fever, and Sore Throat

**Published:** 1894-02-03

**Authors:** 


### Current Medical Topics.

### DIPHTHERIA, SCARLET FEVER, AND SORE THROAT.

One effect of the Notification Act is to necessitate exactness in diagnosis. Unfortunately disease declines to be proportionately definite in symptoms, and in regard to the relation between diphtheria, scarlet fever, and sore throat difficulties are constantly arising. Are cases of scarlet fever with membranous exudation the result of a simultaneous double infection, and are the sore throats which prevail along with diphtheria only abortive examples of this disease? Questions of this sort are constantly arising, and for notification purposes have to be answered. Under these circumstances the paper read at a recent meeting of the Clinical Society by Drs. Washbourn and Goodall on "Cases of Membranous Inflammation of the Throat during Scarlet Fever" was of great interest, although taken by itself, not tending to lessen the complexity of the subject. They stated as the result of bacteriological examination in several cases, and clinical observation in a much larger number, that the membrane which often shows itself on the throat in an early stage of scarlet fever is not true diphtheria, whereas true diphtheria with its full clinical history, including its tendency to affect the larynx and its paralytic sequelæ, is common enough in the later and convalescent stages of the disease. This is fully in accord with the observations of Dr. Klein, recorded in the last report of the medical officer to the Local Government Board, where the same fact is stated, viz., that the membrane observed in the early stage of scarlet fever is not dyphtheritic, while that occurring later is. We have here, then, a distinct rebound from the opinion so largely held a few years ago, in the times of the croup and diphtheria discussions, that all membrane was diphtheria, for now we are told that this tough membrane, although it spreads in a continuous layer and can be detached in masses, still is not diphtheritic, microscopic examination showing that the micro-organisms it contains are distributed differently from those in true diphtheria, extending down into the tissues instead of being almost confined to the superficial layers, and that they are cocci rather than bacilli, cultures also showing an absence of the bacillus diphtheriæ. The membrane of early scarlet fever also does not tend to spread to the larynx, even though inflammation or ulceration may occur there, and paralysis is not found as a sequel to it.

The chances are indeed always against double infections, and experience teaches that as a rule a specific disease can hold its own during at any rate the acute stage. When, however, active symptoms are subsiding, leaving only an injured throat, diphtheria finds its opportunity. Some other observations have recently been published which bear upon this point. Dr. Dowson, in last month's *Medical Chronicle*, puts forward the proposition that scarlet fever is a disease having a primary local lesion situated in the tonsils; that, in fact, it is a disease of these parts and the associated lymphatic glands, the general symptoms being caused by the absorption of toxines produced by the local microbic growth; but he specially points out that scarlet fever always leaves a change in the tonsils, so that after an attack they remain in an abnormal condition.

Here comes in the interest of the view which has of late years taken form in men's minds, that diphtheria is a disease of diseased throats rather than of healthy ones, and which Dr. Thorne Thorne, speaking at the Sanitary Institute a few weeks ago, expressed by saying that "he had formed the opinion that diphtheria never laid hold of a healthy throat." This is probably the key to the position. If diphtheria occurs as a sequel to scarlet fever, or to measles, in consequence of these diseases leaving a morbid condition of throat on which it can take root, why should it not also grow on the morbid condition produced by common inflammatory sore throat? The habit has grown up of late of looking on all the sore throats which prevail so largely at diphtheria times as themselves potentially cases of diphtheria, but seeing that they show no local sign, and are followed by no specific sequelæ, it seems more reasonable to suppose that they bear the same relation to diphtheria as do the morbid throats left by scarlet fever, a soil on which it can grow. In this relation the observations of Dr. Caiger, published in the last report of the Metropolitan Asylums Board, definitely associating the occurrence of post-scarletinal diphtheria with overcrowding and deficiency of floor-space, are of much interest, as bringing these cases into line with those of diphtheria which develop when sore throats are aggregated in school buildings.

As the result then of many observations by different people, working at quite different aspects of the subject, opinion seems drifting towards the idea that diphtheria is a definite infective disease, due to a specific organism, but one the suitable soil for which lies in unhealthy throats. This preceding unhealthiness may be catarrh, or scarlet fever, or that infectious inflammatory sore throat which is so common when diphtheria is about, and on the tide of which the diphtheria epidemic seems to be swept along. Doubtless by rapidly repeated growth on suitable soil the bacillus may undergo a "progressive development of infectiveness," and so may sometimes gain the power of attacking normal throats, but usually diphtheria would seem to be a disease riding on some previous diseased condition. Its prevention then is not to be attained by merely isolating those suffering from it, but by seeking out, and curing as quickly as may be, all those conditions of sore throat which are its special soil, and especially by preventing the aggregation of sore throats of any sort, whether in hospitals or schools. "Sore throats" should be notified and isolated, not necessarily because they are themselves diphtheria, but because they are the soil on which diphtheria grows.

---

Two New York medical men, Dr. M. Wilton Weill and Dr. J. Mount Bleyer, have been experimenting on the influence of the galvanic current in promoting the formation of ozone in the blood. Without giving a definite explanation of what they mean by the term, they have arrived at the conclusion that "animal electricity" is the prime factor in all processes of change of chemical action or otherwise in the living body.

